# Proliferation of Ewing sarcoma cell lines is suppressed by the receptor tyrosine kinase inhibitors gefitinib and vandetanib

**DOI:** 10.1186/1475-2867-8-1

**Published:** 2008-01-04

**Authors:** Mattias K Andersson, Pierre Åman

**Affiliations:** 1 Lundberg Laboratory for Cancer Research, Department of Pathology, Sahlgrenska Academy at Göteborg University, Göteborg, Sweden

## Abstract

**Background:**

Tyrosine kinase inhibitors (TKIs) have gained much attention in recent years as targeted agents for the treatment of a wide range of human cancers. We have investigated the effect of the TKIs gefitinib and vandetanib on tumor cell lines derived from Ewing sarcoma, a highly malignant tumor affecting bone and soft tissue in children and young adults. Gefitinib is an inhibitor of epidermal growth factor receptor tyrosine kinase activity (EGFR) and vandetanib selectively targets vascular endothelial growth factor receptor-2 (VEGFR-2) with additional activity against VEGFR-3, EGFR and RET kinase receptors.

**Results:**

Two Ewing sarcoma cell lines investigated showed high levels of nuclear EGFR expression as well as moderate expression in plasma membrane and cytoplasm. When treated with concentrations of 5 μM and more of either gefitinib or vandetanib, we observed a significant decrease in cell proliferation. However, there were no detectable changes in p44/42 MAPK and Akt-1 phosphorylation, or in the expression of cyclin D1 or c-Myc following gefitinib or vandetanib treatment.

**Conclusion:**

We conclude that Ewing sarcoma tumor cell proliferation is not highly sensitive to inhibition of EGFR signaling alone or the simultaneous inhibition of VEGFR receptors, EGFR and RET kinase. Decreased tumor cell proliferation could be achieved with gefitinib and vandetanib, but only at higher doses where non-specific effects of the compounds may be overriding. As Ewing tumor cells do not seem to depend on EGFR and VEGFR pathways for survival, other key factors in the cellular signaling of Ewing sarcoma should be targeted in order to obtain a potent therapeutic response.

## Background

The Ewing sarcoma family of tumors (ESFTs) is a group of highly malignant tumors affecting bone and soft tissue in children and young adults. ESFT are characterized by reciprocal chromosomal translocations involving the *EWSR1 *gene and members of the *ets *gene family. Multimodal treatment cures about 60% of patients with a localized tumor; however, patients not responsive to therapy, those with detectable metastases at diagnosis and patients with recurrent disease, have a much poorer prognosis, with a cure rate of less than 20%. New therapeutic regimens are therefore needed to treat these diseases [1,2].

Tyrosine kinases are a family of enzymes that are important mediators of signal transduction. They function by selectively phosphorylating target proteins on specific tyrosine residues, using ATP as a substrate. The observation that tyrosine kinases are frequently mutated or otherwise deregulated in human malignancies has led to the emergence of these enzymes as important therapeutic targets in cancer. This has prompted the development and clinical application of tyrosine kinase inhibitors (TKIs) across a broad spectrum of malignancies. TKIs are small organic molecules that inhibit the kinase activity of specific tyrosine kinases by blocking their ATP binding pocket[3].

The aim of this study was to investigate the antiproliferative effect of the TKIs gefitinib (IRESSA™, ZD1839) and vandetanib (ZACTIMA™, ZD6474) on two Ewing sarcoma cell lines. Gefitinib, an inhibitor of epidermal growth factor receptor (EGFR) tyrosine kinase activity, is approved in certain markets for the treatment of non-small cell lung cancer (NSCLC) [4-6]. In addition, a prior study showed partial response for gefitinib in one patient diagnosed with recurrent Ewing sarcoma [7]. Vandetanib, a selective inhibitor of vascular endothelial growth factor receptor (VEGFR), EGFR and RET receptor kinase signaling [8-10], has recently entered Phase III clinical development in NSCLC.

Earlier studies have shown that treatment of tumor cells with TKIs targeting the EGFR family of receptors downregulates mitogen-activated protein kinase (MAPK) and phosphatidylinositol-3-kinase (PI3K)-Akt signaling (reviewed in Hynes and Lane [11]). Consequently, we assessed the effects of gefitinib and vandetanib on downstream targets in these pathways [12,13]).

## Results

### EWS TC71 and EWS IOR/CAR cell lines express EGFR

**Figure 1:**
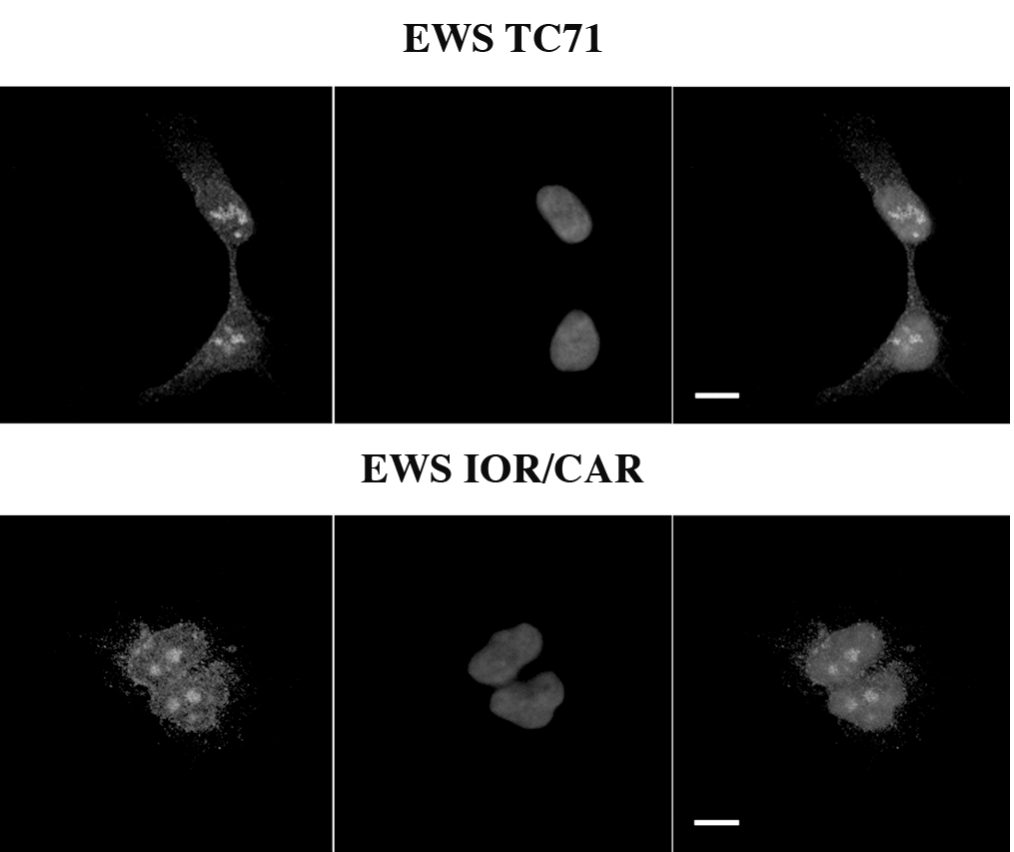
**Ewing tumor cells overexpressing EGFR**. Cells (EWS TC71 and EWS IOR/CAR) were fixed and stained for EGFR using rabbit polyclonal antibodies, which were visualized with goat Cy3-conjugated secondary antibodies. Images show Cy3 fluorescence (*left*), DAPI staining of nuclei (*middle*) and merge (*right*). Images were recorded by laser scanning microscopy. Bars indicate 10 μM.

EGFR expression was confirmed by immunofluorescent imaging (Fig. 1). Both cell lines showed EGFR expression in the plasma membrane and cytoplasm, as well as high levels of nuclear accumulation.

### Gefitinib and vandetanib inhibit growth of Ewing sarcoma cell lines

**Figure 2:**
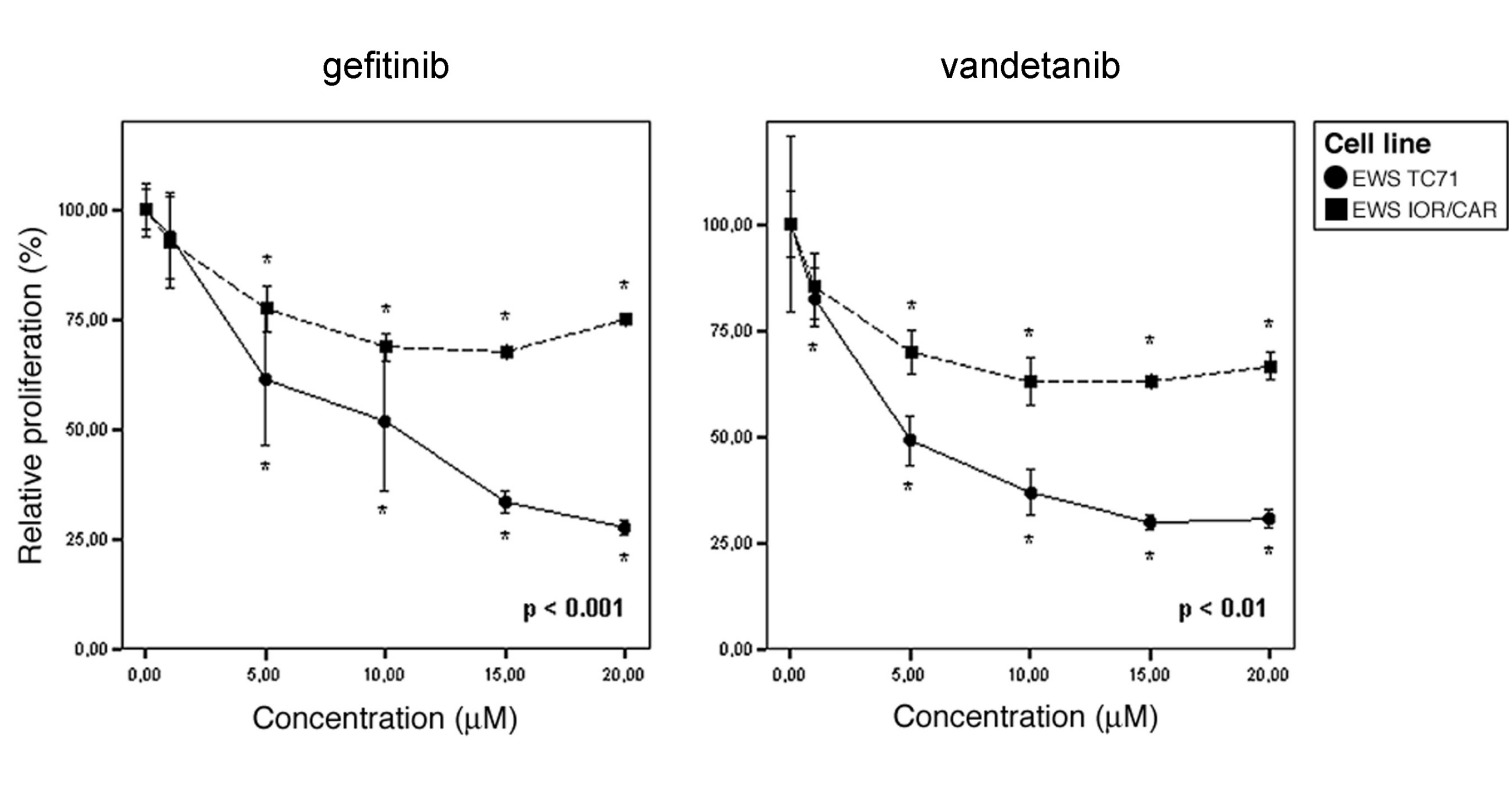
**Proliferation of Ewing tumor cells treated with tyrosine kinase inhibitors for 72 hours**. Cells were treated with indicated concentrations of drugs and relative cell proliferation was assayed in comparison to untreated control cells. Results shown are the means of ten replicates and error bars show 95.0% confidence interval of mean. Asterisks indicate significant inhibition of proliferation at p values indicated in the figures. Data represents one of three experiments yielding similar results.

Growth of the EWS TC71 cell line was markedly inhibited by both drugs with a significant antiproliferative effect observed at 5 μM gefitinib and 1 μM vandetanib (Fig. 2). The IC_50 _values for gefitinib and vandetanib in EWS TC71 were estimated to be ~10 μM and ~5 μM, respectively. The EWS IOR/CAR cell line was less sensitive to TKI treatment but still demonstrated significant growth suppression at 5 μM gefitinib or vandetanib (Fig. 2). IC_50 _values could not be calculated for the EWS IOR/CAR following 72 hours of treatment with 1–20 μM of either drug.

### Gefitinib and vandetanib have no effect on P44/42 MAPK/Akt-1 phosphorylation and cyclin D1/c-Myc expression

**Figure 3:**
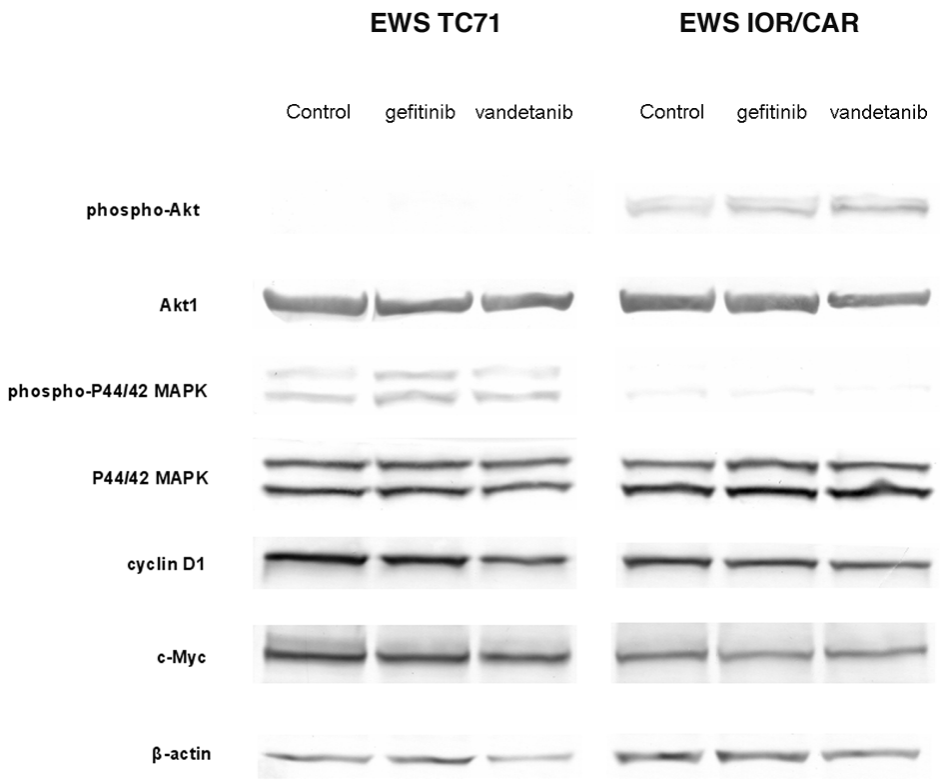
**Effects on MAPK and PI3K-AKT pathways following treatment with tyrosine kinase inhibitors**. Cells were serum starved for 24 hours and then incubated for 1 hour with 10 μM gefitinib or vandetanib. Following this, cells were stimulated with medium containing 10% fetal bovine serum for 4 hours, still in the presence of 10 μM gefitinib or vandetanib. Control cells were treated with vehicle (DMSO) only. Cell extracts were analyzed by western blot using β-actin as a loading control.

Deregulation of cellular signaling in cells treated with the two tyrosine kinase inhibitors was analyzed by studying p44/42 MAPK and Akt-1 phosphorylation as well as cyclin D1 and c-Myc protein levels. Phosphorylated Akt-1 was detected in the EWS IOR/CAR cell line but not in EWS TC71 cells whereas phosphorylated p44/p42 MAPK was detected in the EWS TC71 cell line but only at low levels in EWS IOR/CAR cells (Fig. 3). The phosphorylation status of both proteins was unchanged following incubation with 10 μM gefitinib or vandetanib (Fig 3.) Neither cyclin D1 nor c-Myc expression patterns were altered in drug treated cells compared with untreated control cells (Fig. 3).

## Discussion

In this study, the antiproliferative effect of gefitinib and vandetanib was investigated in two Ewing sarcoma cell lines. We initially examined EGFR expression in both cell lines and found that EGFR is present as aggregates in the tumor cell nuclei as well as localized to the plasma membrane and cytoplasm. Previous studies have shown that nuclear accumulation of EGFR correlates with poor prognosis in breast cancer and oropharyngeal squamous cell carcinomas [14,15]. The aberrant nuclear EGFR expression in our cell lines was taken as confirmation that the cells used in this study were appropriate models for investigating EGFR targeted treatment.

Proliferation of the tumor cell lines was significantly inhibited in the presence of gefitinib or vandetanib. However, the IC_50 _values obtained with EWS TC71 (~10 μM and ~5 μM for gefitinib and vandetanib, respectively) were markedly higher than the nanomolar concentrations of gefitinib and vandetanib observed to inhibit EGFR and VEGFR-2 kinase activity *in vitro *[5,10]. This suggests that the inhibition of EGFR alone or combined inhibition of EGFR, VEGFR2 and RET did not have potent effects on cell proliferation with the culture conditions used in the present study. At high concentrations of gefitinib or vandetanib, significant inhibition of Ewing sarcoma tumour cell growth (>1 μM vandetanib, >5 μM gefitinib) was acheieved but this inhibition is most likely due to off-target effects. The EWS IOR/CAR cell line was less sensitive to both gefitinib and vandetanib and IC_50 _values could not be determined following drug treatment of this cell line in the concentration range tested. Differential sensitivity to EGFR inhibitors have been previously reported for a large panel of cancer cell lines with varying degrees of EGFR expression [16].

Deregulation of cellular signaling in cells treated with gefitinib and vandetanib was analyzed by studying p44/42 MAPK and Akt-1 phosphorylation following serum stimulation in conjunction with drug treatment. We also investigated cyclin D1 and c-Myc protein levels under the same conditions since both cyclin D1 and c-Myc are important downstream targets in EGFR signaling [12,13]. Neither gefitinib nor vandetanib had an effect on p44/42 MAPK and Akt-1 phosphorylation. Surprisingly, discrepancies in the levels of phosphorylated p44/42 MAPK and Akt-1 were observed between the cell lines, with the EWS TC71 cell line lacking phosphorylated Akt-1 and the EWS IOR/CAR line showing only small amounts of phosphorylated p44/42 MAPK. Thus, there are clearly differences between the cell lines with respect to activation of, and dependence on, MAPK and PI3K-AKT pathways. These observations contrast with earlier findings demonstrating constitutive activation of the MAPK and PI3K-AKT pathways in Ewing sarcoma cell lines [17]. Furthermore, we did not detect any changes in cyclin D1 or c-Myc levels when cells were incubated with gefitinib or vandetanib. However, the incubation time used in this study might be too short to elicit a change in the protein levels of cyclin D1 and c-Myc. We presume that the treatment with TKIs would rapidly prevent phosphorylation of MAPK and PI3K-AKT but the activation of these proteins does not appear to be dependent on EGFR- and VEGFR-mediated signaling in the cell lines used in this study. Several deregulated growth factor circuits have been described for Ewing sarcoma cells in the literature such as signaling through the insulin-like growth factor I receptor [18], by human gastrin-releasing peptide [19] and basic fibroblast growth factor [20], as well as platelet derived growth factor[21,22]. Any of these pathways could be involved in the activation of MAPK and PI3K-AKT signaling in the cells studied.

Inhibition of EGFR or VEGFR-2 signaling by gefitinib or vandetanib is inadequate to inhibit tumor cell proliferation *in vitro *other than through unspecific toxicity. Hence, the survival of Ewing sarcoma cells in culture does not seem to depend on EGFR or VEGFR signaling alone. Other growth stimulatory pathways that are essential for tumor cell viability must be targeted in order to obtain a therapeutic response, may it be simultaneously with EGFR/VEGFR-2 inhibition or by other means. Previous work has reported that gefitinib in combination with standard cytotoxic agents potentiates the antitumor activity and results in prolonged survival of mice compared to gefitinib or cytotoxic agents alone [23]. Moreover, studies have shown that response to gefitinib and vandetanib correlate with mutation status of EGFR [24-27], the status of which is unknown for the cell lines used in this study. Although, no mutations were found in the *EGFR *gene in the one patient treated with gefitinib showing partial response [7].

## Conclusion

We conclude that the sole inhibition of EGFR or VEGFR-2 signaling in Ewing sarcoma cell lines is not sufficient to inhibit tumor cell proliferation other than through unspecific toxicity. Thus, a deeper understanding of the specific factors required for Ewing tumor cell proliferation, the sensitivity of different subtypes, and which of these are inhibited by combinations of drug treatments, will further aid the means of targeting the disease from multiple standpoints and in the process avoiding acquired resistance.

## Methods

### Cell lines

EWS TC71 (expressing EWS-FLI1) and EWS IOR/CAR (expressing EWS-ERG) cell lines were kind gifts from Dr. Katia Scotlandi (Laboratory of Oncologic Research, Orthopaedic Rizzoli Institute, Bologna, Italy). Cells were maintained in IMDM medium (PAA Laboratories GmbH, Austria) supplemented with 10% fetal bovine serum (FBS), penicillin (50 U/ml) and streptomycin (50 μg/ml) at 37°C in 5% CO_2_.

### Reagents

Gefitinib and vandetanib were generously provided by AstraZeneca Pharmaceuticals (Macclesfield, UK). Gefitinib and vandetanib were dissolved in dimethylsulfoxide (DMSO) at stock concentrations of 10 mM and 50 mM, respectively, and stored at -20°C. Drugs were further diluted in growth medium prior to experiments.

### Immunofluorescence

Approximately 2 × 10^5 ^tumor cells were seeded and grown in Lab-Tek flaskettes (Nalge Nunc International) for two days and then fixed in 3.7% formaldehyde in phosphate-buffered saline (PBS). Fixed cells were stained for EGFR with a rabbit polyclonal antibody (sc-03, Santa Cruz Biotechnology) and visualized using goat anti-rabbit Cy3-conjugated secondary antibodies (PA43004, Amersham Biosciences). Slides were mounted in ProLong Gold Antifade with DAPI (Molecular Probes) and imaged using a Zeiss LSM510 META confocal microscope.

### Proliferation assay

Tumor cells (~5 × 10^2^) were seeded in each well of black walled/clear bottom 96-well plates (BD Falcon) pre-treated with Collagen R (Serva Electrophoresis) and allowed to adhere overnight. The following day, gefitinib or vandetanib (0 to 20 μM) were added; a total of 10 replicates were used for each concentration. Control cells were incubated with equivalent volumes of DMSO. After 72 hours, cells were subjected to multiple freeze-thaw lyses at -84°C and 37°, respectively. Cell proliferation relative to untreated control cells was assayed with a CyQuant Cell Proliferation Assay Kit (Molecular Probes) using a SpectraMaxGeminiXS microplate reader (Molecular Devices). The IC_50 _of the drugs was defined as the concentration required for reducing the cell number to 50% of the control.

### Western blotting

Approximately 5 × 10^5 ^tumor cells were seeded and grown on Cell+ tissue culture dishes (Sarstedt) pre-treated with Collagen R (Serva Electrophoresis) and allowed to adhere over night. The following day, cells were incubated with serum-free medium for 24 hours and then treated with 10 μM of either gefitinib or vandetanib for 1 hour in the same medium. Controls were treated with corresponding volumes of vehicle (DMSO). Cells were then stimulated for 4 additional hours in medium containing full serum (10% FBS), still in the presence of gefitinib or vandetanib (10 μM). Following stimulation, cells were scraped off and lysed in radioimmunoprecipitation assay (RIPA) buffer containing 1× Complete Mini Protease Inhibitor (Roche), 50 mM NaF and 1 mM heat-activated Na_3_VO_4_. Samples were run on NuPage 4–12% Bis-Tris gels (Invitrogen) using MOPS running buffer and blotted onto PVDF membranes (Immobilon). Blots were stained with antibodies for p44/42 MAPK (137F5, Cell Signaling Technology), phospho-p44/42 MAPK (20G11, Cell Signaling Technology), Akt1 (2H10, Cell Signaling Technology), phospho-Akt (587F11, Cell Signaling Technology), cyclin D1 (M7155, DakoCytomation), c-Myc (sc-40, Santa Cruz Biotechnology) and β-actin (sc-8432, Santa Cruz Biotechnology). Membranes were incubated with alkaline phosphatase-conjugated secondary antibodies (D 0306 and D 0314, DakoCytomation) and visualized with BCIP/NBT tablets (Sigma).

### Statistical methods

Independent samples t-test was used to detect significant differences in proliferation between TKI-treated cells and untreated control cells. All statistical tests were two-tailed.

## Competing interests

The author(s) declare that they have no competing interests.

## Authors' contributions

MKA designed and performed the experiments, carried out the statistical analysis and wrote the manuscript. PA conceived of the study and revised the manuscript. All authors read and approved the final manuscript.
